# Roles of Macrophage Polarization and Macrophage-Derived miRNAs in Pulmonary Fibrosis

**DOI:** 10.3389/fimmu.2021.678457

**Published:** 2021-08-13

**Authors:** Amit Kishore, Martin Petrek

**Affiliations:** ^1^Department of Pathological Physiology, Faculty of Medicine and Dentistry, Palacky University, Olomouc, Czechia; ^2^Accuscript Consultancy, Ludhiana, India; ^3^Institute of Molecular and Translational Medicine, Faculty of Medicine and Dentistry, Palacky University, Olomouc, Czechia; ^4^Departments of Experimental Medicine, and Immunology, University Hospital Olomouc, Olomouc, Czechia

**Keywords:** macrophage plasticity, M1/M2 polarization, MicroRNAs, exosomes, pulmonary fibrosis

## Abstract

This mini-review summarizes the current evidence for the role of macrophage activation and polarization in inflammation and immune response pertinent to interstitial lung disease, specifically pulmonary fibrosis. In the fibrosing lung, the production and function of inflammatory and fibrogenic mediators involved in the disease development have been reported to be regulated by the effects of polarized M1/M2 macrophage populations. The M1 and M2 macrophage phenotypes were suggested to correspond with the pro-inflammatory and pro-fibrogenic signatures, respectively. These responses towards tissue injury followed by the development and progression of lung fibrosis are further regulated by macrophage-derived microRNAs (miRNAs). Besides cellular miRNAs, extracellular exosomal-miRNAs derived from M2 macrophages have also been proposed to promote the progression of pulmonary fibrosis. In a future perspective, harnessing the noncoding miRNAs with a key role in the macrophage polarization is, therefore, suggested as a promising therapeutic strategy for this debilitating disease.

## Introduction

Pulmonary fibrosis (PF) is a progressive, irreversible and lethal lung disease and has remained a challenge for clinicians and researchers. The tissue injury accompanied by cellular inflammation in the lungs drives fibrotic response and thus, plays a crucial role in the pathogenesis of fibrosis. Inflammatory cells release TGF-β, the key regulator of several profibrotic cytokines/chemokines, their receptors/subunits, and growth factors inducing epithelial-mesenchymal transition (EMT) (1–3). The pro-inflammatory and profibrotic cytokines involved in PF promote inflammation and irreversible damage to lung architecture with the loss of alveolar-capillary barrier basal membrane leading to persistent fibrosis (4). These pathogenic factors for PF have further been reported as associated with genetic factors including gene variants and non-coding regulatory microRNAs (3–7).

The tissue‐resident macrophages (M0) are versatile cells that exhibit a high degree of plasticity represented by classically activated M1 (pro-inflammatory) or alternatively activated M2 (anti-inflammatory/pro-fibrotic) macrophages (8). The macrophage polarization is extremely variable and switching of one “activation-type” to another, stimulated by appropriate factors or tissue microenvironment, is a rapid and reversible process. The cross-talk between macrophages and the microenvironment regulates tissue regeneration, flagged with key surface markers in both pro-regenerative and profibrotic environments (9).

Thus, besides the development of tissue homeostasis, these cells are suggested with sequential roles in both induction and resolution of inflammation. The M0 macrophages can polarize to M1 or M2 (M2a, M2b, M2c, and M2d) in response to different activators, such as LPS/IFNγ and IL4/IL13, respectively (10). The M1 macrophage phenotype can also be stimulated without the presence of lymphocytes, for example, by inflammatory cytokines and microorganism‐derived molecules (10–13). The anti-inflammatory or immune-compromised state associated with the M2 macrophage phenotype is also supported with suppressed multiple interferon-associated pathways as one of the most prominent signals common among all M2-polarizing stimuli (14). The M1 macrophage can readily repolarize to M2a (stimulated with reduced IL10 and TNFα, and increased Ym1 level) and M2b (increased IL10 and reduced TNFα) subpopulations (13). Similarly, macrophage M2b can convert to other M2 subtypes in response to different stimuli (15). The different approaches to macrophage polarization have been associated with both the demerits and benefits influencing their utility for specific tissues. Where possible, future therapeutic approaches are suggested to consider tailoring of strategy towards the formation of a specific tissue-microenvironment, as well as promotion of specific disease-associated cell subsets, to improve efficacy and minimize off-target effect (11).

In deciphering pathomechanisms of lung fibrosis, pulmonary macrophages have been implicated with a key role in the fibrogenic process. The PF has been proposed to be regulated by macrophage plasticity (M1/M2 polarization) with an immunogenic signature network of chemokines such as MCP-1, MIP-1α, CCL18, and cytokines such as TNFα, TGFβ1, and their respective signaling pathways (16–18). The ﻿pro-inflammatory M1 macrophage polarization with overexpression of iNOS, TNFα, IL1, IL6, IL12, IL23, MCP-1, and IFN*γ* is associated with inflammation, antitumoral functions and graft rejection (**Table 1**). The anti-inflammatory M2 macrophage polarization, characterised by overexpression of signature proteins such as TGFβ1, IL10, Arginase1, CD204, CD206, VEGF, Ym1, PDGF, MMPs, and IL4Ralpha, was associated with immune regulation, matrix deposition, tissue remodelling, protumoral functions, and graft acceptance (17, 19). The antitumoral and protumoral role of M1 and M2 macrophages, respectively, are further supported with longer survival outcomes among patients with a high M1/M2 ratio in cancers such as ovarian (20, 21) and breast (22) cancer.

**Table 1:** Macrophage subtypes, its activators and implication of cytokines and chemokines in functional response that is also regulated by microRNAs-mediated macrophage plasticity.

M1 and M2 subtypes	Polarization activators	Cytokines and chemokines	Functional response	MicroRNAs expression and its role in M1/M2 macrophage polarization
M1	LPS, IFNγ, TNFα and GM-CSF	Cytokines: TNFα, IL1β, IL6, IL8/CXCL8, IL12, IL23	Th1 response to infection; produces ﻿pro-inflammatory molecules, including TNFα and IL1, IL6, IL12, IL23	↑: miR-21 (M1﻿⊣; M2→), ﻿-33 (M1﻿→; M2⊣), -34a (M1﻿→; M2⊣), -101 (M1﻿→; M2⊣), -125b-5p (M1﻿→; M2⊣), -146b (M1﻿⊣), -155 (M1﻿→; M2⊣), ﻿-342-5p (M1﻿→)
		Chemokines: CCL2, CCL3, CCL4, CCL5, CCL8, CCL9, CCL10, CCL11, CXCL1		↓: ﻿miR-125b-5p (M1﻿⊣), let-7e (M1⊣)
M2a	﻿IL4, IL13	Cytokines: IL10, TGFβ, IL1Rα	﻿Th2 cells, eosinophils, basophils, and macrophages produce IL4. Facilitation of parasite encapsulation	↑: ﻿miR-124 (M1﻿⊣; M2→), -125a-5p (M1﻿→), -135b (M2﻿↑), -146a (M1﻿⊣; M2→), ﻿let-7c (M1﻿⊣; M2→), ﻿-511-3p (M2→), ﻿-378-3p (M2→), ﻿-223 (M1﻿⊣; M2→), ↓: miR-140 (M2→)
		Chemokines: CCL17, CCL22, CCL24		
M2b	Immune complexes plus TLR or IL1R ligands	Cytokines: TNF, IL1β, IL6, IL10	﻿Immunoregulation with up-regulated IL10 and antigen presentation (MHC II, CD86), and down-regulated IL12	
		Chemokines: CCL1		
M2c	﻿IL10, TGFß1 and glucocorticoids	Cytokines: IL10, TGFβ	Tissue remodelling and extracellular matrix production	
M2d	﻿IL6 and adenosine	–	Tumour-associated immune regulation	

TNF-α, Tumour necrosis factor-α; IFN-*γ*, Interferon-*γ*, LPS, lipopolysaccharide; GM-CSF, granulocyte-macrophage colony stimulation factor; ﻿IL, interleukin; miR, microRNA.

Symbols for MicroRNA expression level: ↑, up-regulation; ↓, down-regulation.

Symbols for M1/M2 macrophage polarization: →, progression; ⊣, inhibition.

## Role of Lung Macrophage Polarization in Pulmonary Fibrosis

Macrophages are innate immune cells with antimicrobial phagocytic activity and also play a key role in the pathogenesis of fibrotic disease of pulmonary interstitium. Macrophages are involved at all stages of lung injury and repair, and can promote as well as inhibit fibrosis (16, 23). Airway lumen-based Alveolar macrophages (AM) with surface markers CD11b^low^ CD11c^++^ CD169^+^, and lung parenchymal interstitial macrophages (IM) with﻿ CD11b^+^ CD11c^lo^ CD169^-^ are the two major distinct macrophage populations contributing to lung homeostasis (24). During the development processes of tissue injury and inflammatory reaction and their subsequent progression to PF, AM and IM are polarized to different cell phenotypes - M1 and M2 macrophages, respectively (16, 25). During tissue damage and the early inflammatory phase, activation of M1 macrophages clears the pathogenic microorganisms and promotes inflammation through extracellular matrix degrading ﻿matrix metalloproteases (MMPs) and pro-inflammatory cytokines. ﻿The active cytokine milieu, including elevated Th1 cytokines, IL2, IFNγ, and TNFα, is believed to drive the classical pro-inflammatory (M1) macrophage activation, while a proportion of anti-inflammatory M2 macrophages tends to be higher in other types of interstitial lung diseases (ILDs), including idiopathic pulmonary fibrosis (IPF) (26).

**Figure 1:**
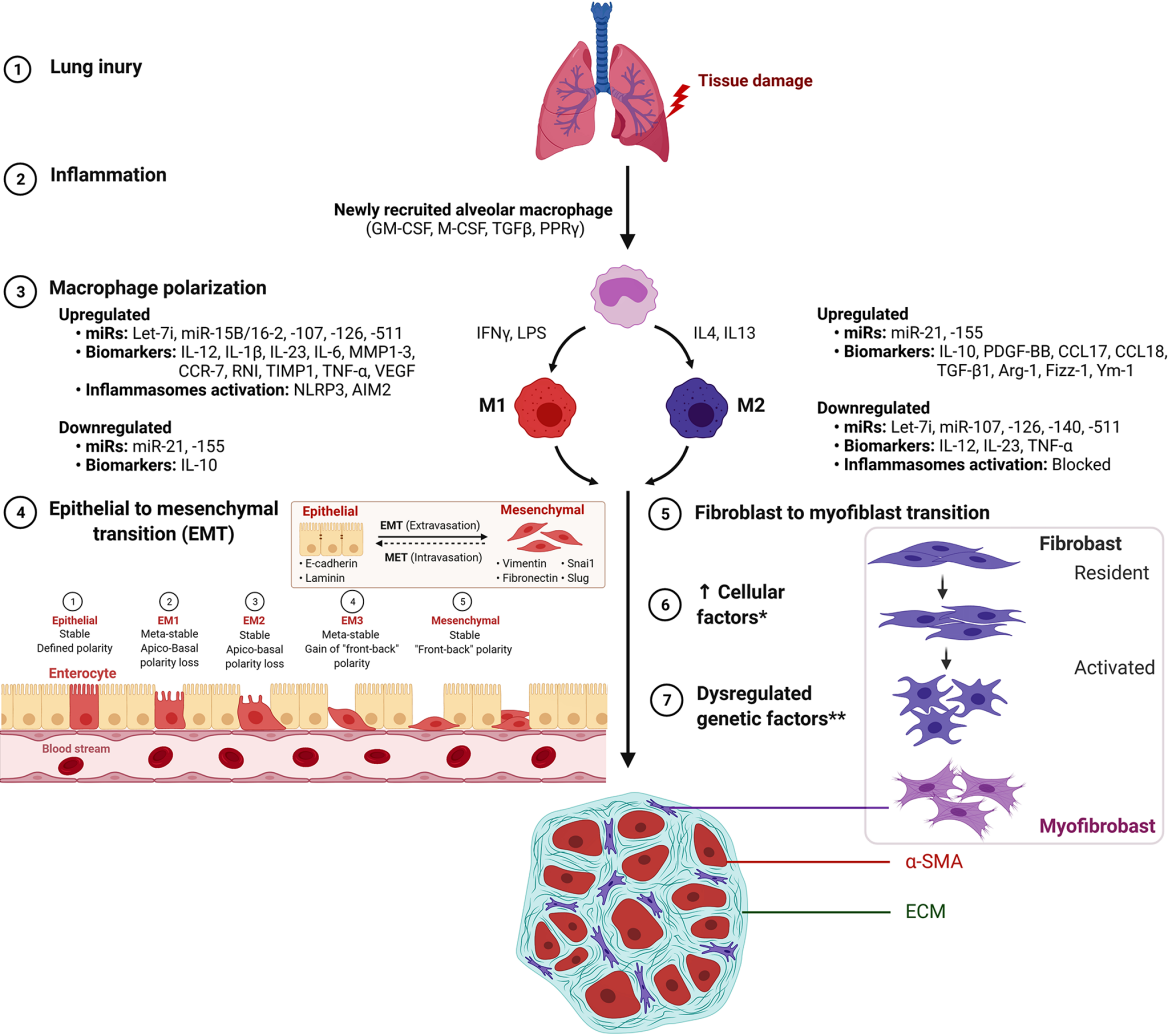
M1 macrophage and M2 macrophage polarization during the development of pulmonary fibrosis. *Increased cellular factors include proliferation, α-smooth muscle actin (α-SMA), matrix factors, collagen, growth factors and cytokines. **Dysregulated genetic factors include upregulated miR-21 and miR-155 and down-regulated Let-7i, miR-107, mir-126, miR-140 and miR-511. Figure created with BioRender. *AIM2*, absent in melanoma 2; *ARG1*, Arginase 1; *Fizz1/RETNLB*, resistin like beta; *Ym1/Chil3*, chitinase-like 3.

The enhanced M2 macrophage polarization has been suggested to inhibit the inflammatory reaction and/or directly regulate the development and progression of fibrotic lung diseases ﻿through the production of chemokines, MMPs, tissue inhibitor of metalloproteinases (TIMPs), and fibronectin as well as, the capability of M2 to differentiate into fibrocyte-like cells that express collagen (27–30). Among ILDs, an increased proportion of M2 macrophages has been observed in granulomas of patients with sarcoidosis as compared with tuberculous granulomas (31). It still needs to be established if a higher proportion of M2 macrophages identifies a profibrotic mechanism inherent to the pathogenesis of sarcoidosis rather than as a part of a generalized wound-healing mechanism to lung inflammation and injury (30, 31). Further, activated macrophages secrete cytokines that attract and stimulate proliferation, promote survival and migration of fibroblast mediated by platelet-derived growth factor (PDGF) (32). In a recent study, inhibition of M2 macrophage polarization has been shown to inhibit bleomycin-induced IPF in rats (33). Similarly, Wang et al. reported that treatment with microcystin-leucine arginine ameliorates PF through suppressed CD206^+^ M2-like macrophage polarization by blocking EMT and fibroblast-myofibroblast transition (FMT), and also substantial reduction of TGFβ1/Smad signaling in rat pulmonary tissues (34). Thus, pro-fibrotic processes such as EMT, FMT, and TGFβ1/Smad signaling represent potential targets in mitigating the development and/or progression of PF (**Figure 1**). Supporting the profibrotic role of M2, a recent study showed attenuation of M2 macrophage infiltration in the lung to significantly protect mice against bleomycin-induced lung injury and fibrosis through suppression of Sart1 by small interfering RNA-loaded liposomes (35).

The macrophage-based pathways implicated in PF majorly include signaling pathways such as TGFβ/Smad (36–39), Wnt/beta-catenin (37, 40–42) and interleukin signaling (43–45). Other signaling pathways reported in a limited number of studies include Lrp5/beta-Catenin (46), MAPK (23), Notch (47), PI3K-AKT-mTOR (48, 49), STAT1 and NF-kappaB (50), IGF-1 receptor (51), 4-1BB (52), NRG-1/ErbB4 (53) and M-CSF/M-CSFR (54). Deciphering the molecular mechanisms of macrophage involved in the development of PF, M2 macrophage was shown to promote EMT through the TGFβ1/Smad2 pathway in bleomycin-induced PF mouse model (39). The PF has been alleviated by pirfenidone through suppressed Wnt/GSK-3beta/beta-catenin and TGFβ1/Smad2/3 signaling pathways (37), and by neohesperidin through TGFβ1/Smad3 inhibition (36); whereas, multiwall carbon nanotubes has been reported to mediate macrophage activation and PF progression through induced TGF-beta/Smad signaling pathway (38).

Macrophage M2 promotes myofibroblast differentiation and is associated with pulmonary fibrogenesis. This process is mitigated by suppressed Wnt/beta-catenin signaling through pirfenidone (37), salinomycin administration (40), and targeted inhibition by PRI-724 (41) and ICG-001 (42). The IL signaling is implicated in PF by IL-4–mediated M2 polarization with elevated Gab 1/2 docking proteins (43), by IL-4Ralpha pathway through crystalline silica exposure (45), or by IL-13 pathway in macrophages induced through sphingosine-1-phosphate receptor-2 (44). Also, activation of p38 MAPK signaling pathway mediated through loss of ﻿﻿a transcription factor Forkhead box M1 (FOXM1) in macrophages was shown to promote PF. Regarding its molecular substance, activation of p38 MAPK pathway in macrophages was reported with the production of pro-fibrotic mediators IL1β, IL6, and TNFα that stimulated fibroblast activation and survival, thus, exacerbating PF (23). Thus, the interplay between M1/M2 macrophage phenotypes has been suggested to play a key role in the development and progression of lung fibrosis (**Figure 1**).

## MicroRNA-Based Regulation of Macrophage Polarization in Immune Response, Inflammation, and Fibrosis

### miRNAs and Their Regulatory Role Towards Macrophage Phenotypes

MicroRNAs (miRNAs) are transcriptional regulators that participate in lung inflammatory responses (5, 55) and are also shown to mediate macrophage polarization. The macrophage subtypes release a various spectrum of cytokines and chemokines that are either pro-inflammatory (M1 phenotypes) and sometimes pro-inflammatory with enhanced tissue destruction, or wound healing and tissue repair (M2 phenotypes), both of which are also regulated through miRNAs (**Table 1**). As an example, miRNA-regulated macrophage polarization is strongly related to miRNA‐124, miRNA‐155, and miRNA‐223. Briefly, higher expression of miRNA‐124 attenuates M1 macrophage, whereas miRNA‐155 promotes M1 and miRNA‐223 depletion also produces M1 polarization (56) (**Supplementary Table S1**). The **Supplementary Table S1** lists a wide spectrum of miRNAs involved in macrophage polarization along with their target proteins and their plausible roles in regulating lung fibrosis.

The regulatory roles of microRNA-mediated macrophage activation and polarization in immune response and inflammation have been extensively reviewed (57–59). This and the following section, therefore, updates the findings of miRNA-mediated macrophage polarization and modulation of pro-inflammatory M1 and/or pro-fibrotic M2 phenotypes in lung disease, in particular, lung fibrosis.

MicroRNA-17, miR-20a, and miR-106a (miR-17/20a/106a) have been shown to effectively regulate alveolar macrophage inflammatory responses such as macrophage infiltration, phagocytosis, and proinflammatory cytokine secretion through targeting leukocyte signal-regulatory protein-α (SIRPα) in both *in-vitro* and *in-vivo* assays (60). The up-regulation of miR-33 in alveolar macrophages exhibited the M1 phenotype with elevated pro-inflammatory cytokines and was demonstrated to promote granuloma formation in a murine model of chronic granulomatous disease, resembling human sarcoidosis pathology, through the suppression of anti-inflammatory lipid transporters (61). An over-expression of macrophage miR-34a has been demonstrated to favour pro-inflammatory M1 phenotype and inhibition of M2 polarization in lipopolysaccharides (LPS) induced acute lung injury (ALI) in mice (62). Similarly, miR-155 was shown to be induced during the macrophage inflammatory response and it orchestrated inflammatory cytokine production in tumour-associated macrophages (TAM). The pro-inflammatory effect of miR-155 has been indicated to promote fibrosis mediated by cross-signaling between macrophages and fibroblasts that governed upregulation of collagen synthesis through TGFβ1 signaling (63). Jaiswal et al., (64) reported overexpression of Let-7c and miR-99a miRNAs in murine bone marrow-derived macrophages (BMDMs) to mitigate Angiotensin-II-induced M1 phenotype activation and to promote M2 phenotype. This inhibition of miR-99a was further shown to reduce ovalbumin-induced Th2 dominance and alleviate allergic airways inflammation (64).

There have also been reports of miR-124 acting to attenuate M1 macrophages as a universal regulator of macrophage into the M2 subtype by decreasing NFκB activity in various subsets of monocytic cells and tissue-resident macrophages including lung macrophages (65, 66). The dysregulation of miR-142-5p and miR-130a-3p was characterised as an important factor governing the polarization of macrophages with higher levels of M2-like phenotypic markers and was associated with airway remodelling in ovalbumin-sensitized mice (67). Another miRNA, miR-146a has been reported to modulate macrophage polarization by inhibiting Notch pathways in RAQ264.7 macrophage cell lines (68). In this context, we observed an elevated level of miR-146a in pulmonary sarcoidosis inflammation (69). Concerning miRNAs involvement in TAM polarization miR-146a-5p, miR-324-5p, miR-223-3p, miR-223-5p, miR-21, miR-125a, miR-130a, and miR-155a were characterized as oncogenic miRNA, while, miR-1207 and miR-320a as a tumour suppressor miRNA in lung cancer including non-small-cell lung carcinoma (NSCLC) (70). These reports thus emphasize the important role of miRNAs in regulating M1/M2 macrophage polarization in lung diseases in general.

### Potential Role of Macrophage-Derived microRNAs in Pulmonary Fibrosis

The microRNA crosstalk influences epithelial-to-mesenchymal and fibroblast-to-myofibroblast transitions implicated in process of macrophage polarization. However, to date, only limited studies have explored the miRNA-based genetic regulation of macrophage polarization and its role in lung fibrosis. An overexpression of let-7c was reported in alveolar macrophages from fibrotic lungs in a bleomycin-induced mouse model as compared with normal lungs, and thus, indicated upregulation of let-7c in macrophages to mitigate M1 phenotype while promoting M2 phenotype polarization (71). Duru et al., (72) reviewed the miRNA-based regulation of macrophage polarization with M2 predominant population in radiation-induced lung fibrosis (RILF) and characterized miR-21 and miR-155 as pro-fibrotic, while let-7i, miR-107, mir-126, miR-140, and miR-511 as anti-fibrotic (72). The IL4 and IL13 induced increased expression of miR-142-5p and downregulated miR-130a-3p transcripts were reported to regulate macrophage profibrogenic expression in tissue samples of patients with IPF (73). The macrophage miR-155 was reported to promote lipopolysaccharide-induced ALI in mice and rats (74). Similarly, miR-155^−/−^ in murine lung macrophages and fibroblasts, and in human IPF lung fibroblasts was implicated in exacerbated pathogenic PF (75). In another study, miR-140 was reported with a key protective role against RILF by inhibiting myofibroblast differentiation and inflammation, and its loss was suggested to induce lung fibrosis through reprogramming fibroblasts and M2 macrophages (76). The role of non-coding RNAs in modulating macrophage phenotypic plasticity and functional heterogeneity among different fibrotic diseases has been recently reviewed (77). These reports further highlight the plausible role of macrophage-derived microRNAs in PF.

### Macrophage-Derived Exosomal miRNAs Mediate Pulmonary Fibrosis

Exosomes are cell-derived vesicles produced by several cell types that function in signaling between cells. Exosomes carry a variety of different biomolecules, such as cytokines and microRNAs, and their content may vary from progenitor or target cells. Exosomal miRNAs have also been implicated in interstitial lung diseases including pulmonary sarcoidosis (55) and IPF (78). Recently, interest has also been gained to decipher the role of macrophage-derived exosomal microRNA (miRNA) in lung fibrosis. Exosomal miRNA-328 from M2 macrophages was shown to enhance pulmonary interstitial fibroblast proliferation and promote the progression of PF in a rat model (78). Besides, macrophage-derived exosomes have been recently suggested to mitigate PF progression *via* delivery of antifibrotic miR-142-3p to alveolar epithelial cells and lung fibroblasts by repressing transforming growth factor β receptor 1 (TGFβ-R1) (79). Another study demonstrated that miRNAs contained in alveolar epithelial type-I cells derived-EVs are actively delivered into alveolar macrophages, subsequently promoting inflammasome activation, neutrophil recruitment, and M1-macrophage polarization and thus endorse pro-inflammatory responses in bacterial lung infection (80). In ALI, young mesenchymal stem cells-derived extracellular vesicles (MSC-EVs) showed higher expression of miR-223-5p and lower levels of miR-127-3p and miR-125b-5p compared with aging MSC-EVs. Further, inhibition of miR-127-3p and miR-125b-5p in BMDMs was reported to downregulate M1 and thus, supported their role in M1 macrophage polarization (81). Besides, MSC-EVs were reported to mitigate ALI at least partially through the transfer of miR-27a-3p to alveolar macrophages and promoted M2 macrophage polarization (34). MiR-27a-3p was also shown to target NFKB1 and thus, was suggested as a key regulator of M2 macrophage polarization (34). Recently, a study investigated the potential connections between arsenic and epigenetic changes that mediate M2 macrophage polarization in the development of PF and reported arsenite, elevated LncRNA H19, c-Myc, and Arg1 along with decreased let-7a to be associated with PF in mice (82). Another recent study in a mouse model reported MSC−derived exosomal miR−135b to promote M2 polarization of synovial macrophage by targeting MAPK6, thus mitigating cartilage injury (83). Thus, evidence supports the role of M2 macrophage-derived exosomal miRNA in pulmonary interstitial fibroblast proliferation and in promoting the progression of lung fibrosis. This is further supported by MSC−EVs-derived miRNAs that are suggested to mediate M2 macrophage polarization in the development of PF (34). In ALI, young and aging MSC-EVs harbours differentially expressed miRNAs associated with M1/M2 macrophage polarization (81).

## Conclusion

The present minireview summarizes major findings on the role of macrophage polarization in diseases, in particular, PF. The non-coding regulatory miRNAs are also discussed in the context of their modulation of M1/M2 macrophage phenotypes in the development and progression of IPF. Further, exosomal miRNA from M2 macrophages favouring pulmonary interstitial fibroblast proliferation and promoting the progression of PF are also described.

In summary, the regulation of macrophage polarization by miRNA is suggested to represent one of the key pathogenetic factors in the development and progression of PF. Further research focused on distinct levels of these processes will undoubtedly provide updated information. Apart from detailing our current theoretical knowledge, it could be translated into future diagnostic approaches and/or designing novel therapeutic strategies helping to combat IPF, which despite the advancements still constitutes a major debilitating disease.

## Author Contributions

AK and MP contributed to the conception and writing of this review. All authors contributed to the article and approved the submitted version.

## Funding

This work was funded in part from Palacky University and Czech government (IGA PU 2021_014, RVO00098892 and CZ.02.1.01/0.0/0.0/16_019/0000868, ENOCH).

## Conflict of Interest

The authors declare that the research was conducted in the absence of any commercial or financial relationships that could be construed as a potential conflict of interest.

## Publisher’s Note

All claims expressed in this article are solely those of the authors and do not necessarily represent those of their affiliated organizations, or those of the publisher, the editors and the reviewers. Any product that may be evaluated in this article, or claim that may be made by its manufacturer, is not guaranteed or endorsed by the publisher.

## References

[1] Martinez FJ Collard HR Pardo A Raghu G Richeldi L Selman M. Idiopathic Pulmonary Fibrosis. Nat Rev Dis Primers (2017) 3:17074. 10.1038/nrdp.2017.74 29052582

[2] Pardo A Selman M. The Interplay of the Genetic Architecture, Aging, and Environmental Factors in the Pathogenesis of Idiopathic Pulmonary Fibrosis. Am J Respir Cell Mol Biol (2021) 64(2):163–72. 10.1165/rcmb.2020-0373PS 32946290

[3] Barros A Oldham J Noth I. Genetics of Idiopathic Pulmonary Fibrosis. Am J Med Sci (2019) 357(5):379–83. 10.1016/j.amjms.2019.02.009 PMC10538522 31010464

[4] Kishore A Zizkova V Kocourkova L Petrek M. A Dataset of 26 Candidate Gene and Pro-Inflammatory Cytokine Variants for Association Studies in Idiopathic Pulmonary Fibrosis: Frequency Distribution in Normal Czech Population. Front Immunol (2015) 6:476. 10.3389/fimmu.2015.00476 26441981 PMC4585032

[5] Kishore A Borucka J Petrkova J Petrek M. Novel Insights Into miRNA in Lung and Heart Inflammatory Diseases. Mediators Inflamm (2014) 2014:259131. 10.1155/2014/259131 24991086 PMC4058468

[6] Kishore A Zizkova V Kocourkova L Petrkova J Bouros E Nunes H. Association Study for 26 Candidate Loci in Idiopathic Pulmonary Fibrosis Patients From Four European Populations. Front Immunol (2016) 7:274. 10.3389/fimmu.2016.00274 27462317 PMC4939450

[7] Allen RJ Guillen-Guio B Oldham JM Ma SF Dressen A Paynton ML. Genome-Wide Association Study of Susceptibility to Idiopathic Pulmonary Fibrosis. Am J Respir Crit Care Med (2020) 201(5):564–74. 10.1164/rccm.201905-1017OC PMC7047454 31710517

[8] Vasse GF Nizamoglu M Heijink IH Schleputz M van Rijn P Thomas MJ. Macrophage-Stroma Interactions in Fibrosis: Biochemical, Biophysical, and Cellular Perspectives. J Pathol (2021) 254(4):344–57. 10.1002/path.5632 PMC8252758 33506963

[9] Perciani CT MacParland SA. Lifting the Veil on Macrophage Diversity in Tissue Regeneration and Fibrosis. Sci Immunol (2019) 4(40):eaaz0749. 10.1126/sciimmunol.aaz0749 31604845

[10] Tarique AA Logan J Thomas E Holt PG Sly PD Fantino E. Phenotypic, Functional, and Plasticity Features of Classical and Alternatively Activated Human Macrophages. Am J Respir Cell Mol Biol (2015) 53(5):676–88. 10.1165/rcmb.2015-0012OC 25870903

[11] Bart VMT Pickering RJ Taylor PR Ipseiz N. Macrophage Reprogramming for Therapy. Immunology (2020) 163(2):128–44. 10.1111/imm.13300 PMC8114216 33368269

[12] Porcheray F Viaud S Rimaniol AC Leone C Samah B Dereuddre-Bosquet N. Macrophage Activation Switching: An Asset for the Resolution of Inflammation. Clin Exp Immunol (2005) 142(3):481–9. 10.1111/j.1365-2249.2005.02934.x PMC1809537 16297160

[13] Kudlik G Hegyi B Czibula A Monostori E Buday L Uher F. Mesenchymal Stem Cells Promote Macrophage Polarization Toward M2b-Like Cells. Exp Cell Res (2016) 348(1):36–45. 10.1016/j.yexcr.2016.08.022 27578361

[14] Gharib SA McMahan RS Eddy WE Long ME Parks WC Aitken ML. Transcriptional and Functional Diversity of Human Macrophage Repolarization. J Allergy Clin Immunol (2019) 143(4):1536–48. 10.1016/j.jaci.2018.10.046 PMC6451869 30445062

[15] Wang LX Zhang SX Wu HJ Rong XL Guo J. M2b Macrophage Polarization and Its Roles in Diseases. J Leukoc Biol (2019) 106(2):345–58. 10.1002/JLB.3RU1018-378RR PMC7379745 30576000

[16] Liu G Zhai H Zhang T Li S Li N Chen J. New Therapeutic Strategies for IPF: Based on the “Phagocytosis-Secretion-Immunization” Network Regulation Mechanism of Pulmonary Macrophages. BioMed Pharmacother (2019) 118:109230. 10.1016/j.biopha.2019.109230 31351434

[17] Lee KY. M1 and M2 Polarization of Macrophages: A Mini-Review. Med Biol Sci Eng (2019) 2(1):1–5. 10.30579/mbse.2019.2.1.1

[18] Murray PJ. Macrophage Polarization. Annu Rev Physiol (2017) 79:541–66. 10.1146/annurev-physiol-022516-034339 27813830

[19] Mantovani A Locati M. Tumor-Associated Macrophages as a Paradigm of Macrophage Plasticity, Diversity, and Polarization: Lessons and Open Questions. Arterioscler Thromb Vasc Biol (2013) 33(7):1478–83. 10.1161/ATVBAHA.113.300168 23766387

[20] Maccio A Gramignano G Cherchi MC Tanca L Melis L Madeddu C. Role of M1-Polarized Tumor-Associated Macrophages in the Prognosis of Advanced Ovarian Cancer Patients. Sci Rep (2020) 10(1):6096. 10.1038/s41598-020-63276-1 32269279 PMC7142107

[21] Zhang M He Y Sun X Li Q Wang W Zhao A. A High M1/M2 Ratio of Tumor-Associated Macrophages Is Associated With Extended Survival in Ovarian Cancer Patients. J Ovarian Res (2014) 7:19. 10.1186/1757-2215-7-19 24507759 PMC3939626

[22] Suarez-Arriaga MC Mendez-Tenorio A Perez-Koldenkova V Fuentes-Panana EM. Claudin-Low Breast Cancer Inflammatory Signatures Support Polarization of M1-Like Macrophages With Protumoral Activity. Cancers (Basel) (2021) 13(9):2248. 10.3390/cancers13092248 34067089 PMC8125772

[23] Goda C Balli D Black M Milewski D Le T Ustiyan V. Loss of FOXM1 in Macrophages Promotes Pulmonary Fibrosis by Activating P38 MAPK Signaling Pathway. PloS Genet (2020) 16(4):e1008692. 10.1371/journal.pgen.1008692 32271749 PMC7173935

[24] Byrne AJ Maher TM Lloyd CM. Pulmonary Macrophages: A New Therapeutic Pathway in Fibrosing Lung Disease? Trends Mol Med (2016) 22(4):303–16. 10.1016/j.molmed.2016.02.004 26979628

[25] Zhang L Wang Y Wu G Xiong W Gu W Wang CY. Macrophages: Friend or Foe in Idiopathic Pulmonary Fibrosis? Respir Res (2018) 19(1):170. 10.1186/s12931-018-0864-2 30189872 PMC6127991

[26] Wojtan P Mierzejewski M Osinska I Domagala-Kulawik J. Macrophage Polarization in Interstitial Lung Diseases. Cent Eur J Immunol (2016) 41(2):159–64. 10.5114/ceji.2016.60990 PMC4967650 27536201

[27] Byrne AJ Mathie SA Gregory LG Lloyd CM. Pulmonary Macrophages: Key Players in the Innate Defence of the Airways. Thorax (2015) 70(12):1189–96. 10.1136/thoraxjnl-2015-207020 26286722

[28] Gharib SA Johnston LK Huizar I Birkland TP Hanson J Wang Y. MMP28 Promotes Macrophage Polarization Toward M2 Cells and Augments Pulmonary Fibrosis. J Leukoc Biol (2014) 95(1):9–18. 10.1189/jlb.1112587 23964118 PMC3868192

[29] Medbury HJ James V Ngo J Hitos K Wang Y Harris DC. Differing Association of Macrophage Subsets With Atherosclerotic Plaque Stability. Int Angiol (2013) 32(1):74–84. 23435395

[30] Polverino F Balestro E Spagnolo P. Clinical Presentations, Pathogenesis, and Therapy of Sarcoidosis: State of the Art. J Clin Med (2020) 9(8):2363. 10.3390/jcm9082363 PMC7465477 32722050

[31] Shamaei M Mortaz E Pourabdollah M Garssen J Tabarsi P Velayati A. Evidence for M2 Macrophages in Granulomas From Pulmonary Sarcoidosis: A New Aspect of Macrophage Heterogeneity. Hum Immunol (2018) 79(1):63–9. 10.1016/j.humimm.2017.10.009 29107084

[32] Partida-Zavala N Antonio Ponce-Gallegos M Buendia-Roldan I Falfan-Valencia R. Type 2 Macrophages and Th2 CD4+ Cells in Interstitial Lung Diseases (ILDs): An Overview. Sarcoidosis Vasc Diffuse Lung Dis (2018) 35(2):98–108. 10.36141/svdld.v35i2.6691 32476888 PMC7170082

[33] Guo Z Li S Zhang N Kang Q Zhai H. Schisandra Inhibit Bleomycin-Induced Idiopathic Pulmonary Fibrosis in Rats *via* Suppressing M2 Macrophage Polarization. BioMed Res Int (2020) 2020:5137349. 10.1155/2020/5137349 32884941 PMC7455820

[34] Wang J Xu L Xiang Z Ren Y Zheng X Zhao Q. Microcystin-LR Ameliorates Pulmonary Fibrosis *via* Modulating CD206(+) M2-Like Macrophage Polarization. Cell Death Dis (2020) 11(2):136. 10.1038/s41419-020-2329-z 32075954 PMC7031231

[35] Pan T Zhou Q Miao K Zhang L Wu G Yu J. Suppressing Sart1 to Modulate Macrophage Polarization by siRNA-Loaded Liposomes: A Promising Therapeutic Strategy for Pulmonary Fibrosis. Theranostics (2021) 11(3):1192–206. 10.7150/thno.48152 PMC7738894 33391530

[36] Guo J Fang Y Jiang F Li L Zhou H Xu X. Neohesperidin Inhibits TGF-Beta1/Smad3 Signaling and Alleviates Bleomycin-Induced Pulmonary Fibrosis in Mice. Eur J Pharmacol (2019) 864:172712. 10.1016/j.ejphar.2019.172712 31586469

[37] Lv Q Wang J Xu C Huang X Ruan Z Dai Y. Pirfenidone Alleviates Pulmonary Fibrosis *In Vitro* and *In Vivo* Through Regulating Wnt/GSK-3beta/Beta-Catenin and TGF-Beta1/Smad2/3 Signaling Pathways. Mol Med (2020) 26(1):49. 10.1186/s10020-020-00173-3 32448163 PMC7245944

[38] Wang P Nie X Wang Y Li Y Ge C Zhang L. Multiwall Carbon Nanotubes Mediate Macrophage Activation and Promote Pulmonary Fibrosis Through TGF-Beta/Smad Signaling Pathway. Small (2013) 9(22):3799–811. 10.1002/smll.201300607 23650105

[39] Zhu L Fu X Chen X Han X Dong P. M2 Macrophages Induce EMT Through the TGF-Beta/Smad2 Signaling Pathway. Cell Biol Int (2017) 41(9):960–8. 10.1002/cbin.10788 28493530

[40] Hou J Shi J Chen L Lv Z Chen X Cao H. M2 Macrophages Promote Myofibroblast Differentiation of LR-MSCs and are Associated With Pulmonary Fibrogenesis. Cell Commun Signal (2018) 16(1):89. 10.1186/s12964-018-0300-8 30470231 PMC6260991

[41] Okazaki H Sato S Koyama K Morizumi S Abe S Azuma M. The Novel Inhibitor PRI-724 for Wnt/beta-Catenin/CBP Signaling Ameliorates Bleomycin-Induced Pulmonary Fibrosis in Mice. Exp Lung Res (2019) 45(7):188–99. 10.1080/01902148.2019.1638466 31298961

[42] Cao H Wang C Chen X Hou J Xiang Z Shen Y. Inhibition of Wnt/beta-Catenin Signaling Suppresses Myofibroblast Differentiation of Lung Resident Mesenchymal Stem Cells and Pulmonary Fibrosis. Sci Rep (2018) 8(1):13644. 10.1038/s41598-018-28968-9 30206265 PMC6134002

[43] Guo X Li T Xu Y Xu X Zhu Z Zhang Y. Increased Levels of Gab1 and Gab2 Adaptor Proteins Skew Interleukin-4 (IL-4) Signaling Toward M2 Macrophage-Driven Pulmonary Fibrosis in Mice. J Biol Chem (2017) 292(34):14003–15. 10.1074/jbc.M117.802066 PMC5572909 28687632

[44] Zhao J Okamoto Y Asano Y Ishimaru K Aki S Yoshioka K. Sphingosine-1-Phosphate Receptor-2 Facilitates Pulmonary Fibrosis Through Potentiating IL-13 Pathway in Macrophages. PloS One (2018) 13(5):e0197604. 10.1371/journal.pone.0197604 29782549 PMC5962071

[45] Migliaccio CT Buford MC Jessop F Holian A. The IL-4Ralpha Pathway in Macrophages and Its Potential Role in Silica-Induced Pulmonary Fibrosis. J Leukoc Biol (2008) 83(3):630–9. 10.1189/jlb.0807533 18056481

[46] Sennello JA Misharin AV Flozak AS Berdnikovs S Cheresh P Varga J. Lrp5/beta-Catenin Signaling Controls Lung Macrophage Differentiation and Inhibits Resolution of Fibrosis. Am J Respir Cell Mol Biol (2017) 56(2):191–201. 10.1165/rcmb.2016-0147OC 27668462 PMC5359648

[47] Zhang N Yang K Bai J Yi J Gao C Zhao J. Myeloid-Specific Blockade of Notch Signaling Alleviates Murine Pulmonary Fibrosis Through Regulating Monocyte-Derived Ly6c(lo) MHCII(hi) Alveolar Macrophages Recruitment and TGF-Beta Secretion. FASEB J (2020) 34(8):11168–84. 10.1096/fj.201903086RR 32638441

[48] Han B Chu C Su X Zhang N Zhou L Zhang M. N(6)-Methyladenosine-Dependent Primary microRNA-126 Processing Activated PI3K-AKT-mTOR Pathway Drove the Development of Pulmonary Fibrosis Induced by Nanoscale Carbon Black Particles in Rats. Nanotoxicology (2020) 14(1):1–20. 10.1080/17435390.2019.1661041 31502903

[49] Tsitoura E Wells AU Karagiannis K Lasithiotaki I Vasarmidi E Bibaki E. MiR-185/AKT and miR-29a/Collagen 1a Pathways Are Activated in IPF BAL Cells. Oncotarget (2016) 7(46):74569–81. 10.18632/oncotarget.12740 PMC5342687 27769060

[50] Haydar D Cory TJ Birket SE Murphy BS Pennypacker KR Sinai AP. Azithromycin Polarizes Macrophages to an M2 Phenotype *via* Inhibition of the STAT1 and NF-kappaB Signaling Pathways. J Immunol (2019) 203(4):1021–30. 10.4049/jimmunol.1801228 PMC6684391 31263039

[51] Chung EJ Kwon S Reedy JL White AO Song JS Hwang I. IGF-1 Receptor Signaling Regulates Type II Pneumocyte Senescence and Resulting Macrophage Polarization in Lung Fibrosis. Int J Radiat Oncol Biol Phys (2021) 110(2):526–38. 10.1016/j.ijrobp.2020.12.035 PMC8784947 33385497

[52] Lu Y Li C Du S Chen X Zeng X Liu F. 4-1bb Signaling Promotes Alveolar Macrophages-Mediated Pro-Fibrotic Responses and Crystalline Silica-Induced Pulmonary Fibrosis in Mice. Front Immunol (2018) 9:1848. 10.3389/fimmu.2018.01848 30250465 PMC6139304

[53] Vermeulen Z Hervent AS Dugaucquier L Vandekerckhove L Rombouts M Beyens M. Inhibitory Actions of the NRG-1/ErbB4 Pathway in Macrophages During Tissue Fibrosis in the Heart, Skin, and Lung. Am J Physiol Heart Circ Physiol (2017) 313(5):H934–H45. 10.1152/ajpheart.00206.2017 28822966

[54] Joshi N Watanabe S Verma R Jablonski RP Chen CI Cheresh P. A Spatially Restricted Fibrotic Niche in Pulmonary Fibrosis Is Sustained by M-CSF/M-CSFR Signalling in Monocyte-Derived Alveolar Macrophages. Eur Respir J (2020) 55(1):1900646. 10.1183/13993003.00646-2019 31601718 PMC6962769

[55] Kishore A Navratilova Z Kolek V Novosadova E Cepe K du Bois RM. Expression Analysis of Extracellular microRNA in Bronchoalveolar Lavage Fluid From Patients With Pulmonary Sarcoidosis. Respirology (2018) 23(12):1166–72. 10.1111/resp.13364 29956871

[56] Self-Fordham JB Naqvi AR Uttamani JR Kulkarni V Nares S. MicroRNA: Dynamic Regulators of Macrophage Polarization and Plasticity. Front Immunol (2017) 8:1062. 10.3389/fimmu.2017.01062 28912781 PMC5583156

[57] Essandoh K Li Y Huo J Fan GC. MiRNA-Mediated Macrophage Polarization and Its Potential Role in the Regulation of Inflammatory Response. Shock (2016) 46(2):122–31. 10.1097/SHK.0000000000000604 PMC4949115 26954942

[58] Liu G Abraham E. MicroRNAs in Immune Response and Macrophage Polarization. Arterioscler Thromb Vasc Biol (2013) 33(2):170–7. 10.1161/ATVBAHA.112.300068 PMC3549532 23325473

[59] Wu XQ Dai Y Yang Y Huang C Meng XM Wu BM. Emerging Role of microRNAs in Regulating Macrophage Activation and Polarization in Immune Response and Inflammation. Immunology (2016) 148(3):237–48. 10.1111/imm.12608 PMC4913289 27005899

[60] Zhu D Pan C Li L Bian Z Lv Z Shi L. MicroRNA-17/20a/106a Modulate Macrophage Inflammatory Responses Through Targeting Signal-Regulatory Protein Alpha. J Allergy Clin Immunol (2013) 132(2):426–36 e8. 10.1016/j.jaci.2013.02.005 23562609 PMC5882493

[61] Barna BP McPeek M Malur A Fessler MB Wingard CJ Dobbs L. Elevated MicroRNA-33 in Sarcoidosis and a Carbon Nanotube Model of Chronic Granulomatous Disease. Am J Respir Cell Mol Biol (2016) 54(6):865–71. 10.1165/rcmb.2015-0332OC PMC4942222 26641802

[62] Khan MJ Singh P Dohare R Jha R Rahmani AH Almatroodi SA. Inhibition of miRNA-34a Promotes M2 Macrophage Polarization and Improves LPS-Induced Lung Injury by Targeting Klf4. Genes (Basel) (2020) 11(9):966. 10.3390/genes11090966 PMC7563942 32825525

[63] Eissa MG Artlett CM. The MicroRNA miR-155 Is Essential in Fibrosis. Noncoding RNA (2019) 5(1):23. 10.3390/ncrna5010023 PMC6468348 30871125

[64] Jaiswal A Maurya M Maurya P Barthwal MK. Lin28B Regulates Angiotensin II-Mediated Let-7c/miR-99a MicroRNA Formation Consequently Affecting Macrophage Polarization and Allergic Inflammation. Inflammation (2020) 43(5):1846–61. 10.1007/s10753-020-01258-1 32458348

[65] Arora S Dev K Agarwal B Das P Syed MA. Macrophages: Their Role, Activation and Polarization in Pulmonary Diseases. Immunobiology (2018) 223(4-5):383–96. 10.1016/j.imbio.2017.11.001 PMC7114886 29146235

[66] Liang Y Xie J Che D Zhang C Lin Y Feng L. MiR-124-3p Helps to Protect Against Acute Respiratory Distress Syndrome by Targeting P65. Biosci Rep (2020) 40(5):BSR20192132. 10.1042/BSR20192132 32391561 PMC7253404

[67] Shi J Chen M Ouyang L Wang Q Guo Y Huang L. miR-142-5p and miR-130a-3p Regulate Pulmonary Macrophage Polarization and Asthma Airway Remodeling. Immunol Cell Biol (2020) 98(9):715–25. 10.1111/imcb.12369 32524675

[68] Huang C Liu XJ Zhou Q Xie J Ma TT Meng XM. MiR-146a Modulates Macrophage Polarization by Inhibiting Notch1 Pathway in RAW264. 7 Macrophages Int Immunopharmacol (2016) 32:46–54. 10.1016/j.intimp.2016.01.009 26800097

[69] Novosadova E Chabronova A Kolek V Petrek M Navratilova Z. The Serum Expression of Selected miRNAs in Pulmonary Sarcoidosis With/Without Lofgren’s Syndrome. Mediators Inflamm (2016) 2016:1246129. 10.1155/2016/1246129 28050119 PMC5165170

[70] Pirlog R Cismaru A Nutu A Berindan-Neagoe I. Field Cancerization in NSCLC: A New Perspective on MicroRNAs in Macrophage Polarization. Int J Mol Sci (2021) 22(2):746. 10.3390/ijms22020746 PMC7828565 33451052

[71] Banerjee S Xie N Cui H Tan Z Yang S Icyuz M. MicroRNA Let-7c Regulates Macrophage Polarization. J Immunol (2013) 190(12):6542–9. 10.4049/jimmunol.1202496 PMC3679284 23667114

[72] Duru N Wolfson B Zhou Q. Mechanisms of the Alternative Activation of Macrophages and non-Coding RNAs in the Development of Radiation-Induced Lung Fibrosis. World J Biol Chem (2016) 7(4):231–9. 10.4331/wjbc.v7.i4.231 PMC5124699 27957248

[73] Su S Zhao Q He C Huang D Liu J Chen F. miR-142-5p and miR-130a-3p are Regulated by IL-4 and IL-13 and Control Profibrogenic Macrophage Program. Nat Commun (2015) 6:8523. 10.1038/ncomms9523 26436920 PMC4600756

[74] Wang W Liu Z Su J Chen WS Wang XW Bai SX. Macrophage Micro-RNA-155 Promotes Lipopolysaccharide-Induced Acute Lung Injury in Mice and Rats. Am J Physiol Lung Cell Mol Physiol (2016) 311(2):L494–506. 10.1152/ajplung.00001.2016 27371731

[75] Kurowska-Stolarska M Hasoo MK Welsh DJ Stewart L McIntyre D Morton BE. The Role of microRNA-155/Liver X Receptor Pathway in Experimental and Idiopathic Pulmonary Fibrosis. J Allergy Clin Immunol (2017) 139(6):1946–56. 10.1016/j.jaci.2016.09.021 PMC5457127 27746237

[76] Duru N Zhang Y Gernapudi R Wolfson B Lo PK Yao Y. Loss of miR-140 Is a Key Risk Factor for Radiation-Induced Lung Fibrosis Through Reprogramming Fibroblasts and Macrophages. Sci Rep (2016) 6:39572. 10.1038/srep39572 27996039 PMC5172237

[77] Zhou D Wu Y Wang S Li J Luan J. Harnessing Noncoding RNA-Based Macrophage Polarization: Emerging Therapeutic Opportunities for Fibrosis. Immun Inflamm Dis (2020) 8(4):793–806. 10.1002/iid3.341 33080104 PMC7654411

[78] Yao MY Zhang WH Ma WT Liu QH Xing LH Zhao GF. microRNA-328 in Exosomes Derived From M2 Macrophages Exerts a Promotive Effect on the Progression of Pulmonary Fibrosis *via* FAM13A in a Rat Model. Exp Mol Med (2019) 51(6):1–16. 10.1038/s12276-019-0255-x PMC6547742 31164635

[79] Guiot J Cambier M Boeckx A Henket M Nivelles O Gester F. Macrophage-Derived Exosomes Attenuate Fibrosis in Airway Epithelial Cells Through Delivery of Antifibrotic miR-142-3p. Thorax (2020) 75(10):870–81. 10.1136/thoraxjnl-2019-214077 PMC7509395 32759383

[80] Lee H Groot M Pinilla-Vera M Fredenburgh LE Jin Y. Identification of miRNA-Rich Vesicles in Bronchoalveolar Lavage Fluid: Insights Into the Function and Heterogeneity of Extracellular Vesicles. J Control Release (2019) 294:43–52. 10.1016/j.jconrel.2018.12.008 30529727 PMC6372374

[81] Huang R Qin C Wang J Hu Y Zheng G Qiu G. Differential Effects of Extracellular Vesicles From Aging and Young Mesenchymal Stem Cells in Acute Lung Injury. Aging (Albany NY) (2019) 11(18):7996–8014. 10.18632/aging.102314 31575829 PMC6781978

[82] Xiao T Zou Z Xue J Syed BM Sun J Dai X. LncRNA H19-Mediated M2 Polarization of Macrophages Promotes Myofibroblast Differentiation in Pulmonary Fibrosis Induced by Arsenic Exposure. Environ Pollut (2021) 268(Pt A):115810. 10.1016/j.envpol.2020.115810 33162208

[83] Wang R Xu B. TGF-Beta1-Modified MSC-Derived Exosomal miR-135b Attenuates Cartilage Injury *via* Promoting M2 Synovial Macrophage Polarization by Targeting MAPK6. Cell Tissue Res (2021) 384(1):113–27. 10.1007/s00441-020-03319-1 33404840

