# Supplementary material for: Roles of Macrophage Polarization and Macrophage-Derived miRNAs in Pulmonary Fibrosis
Source: Front Immunol. 2021 Aug 13;12:678457. doi: 10.3389/fimmu.2021.678457 (PMC8417529; doi:10.3389/fimmu.2021.678457)
Supplement: Supplementary file 1 [file Table_1.pdf]

Roles of macrophage polarization and macrophage-derived miRNAs in pulmonary fibrosis

Amit Kishore[1,2*], Martin Petrek[1,3,4*]

[1]Department of Pathological Physiology, Faculty of Medicine and Dentistry, Palacky University, Olomouc 77515, Czech Republic

[2]Accuscript Consultancy, Ludhiana 141001, Punjab, India

[3]Institute of Molecular and Translational Medicine, Faculty of Medicine and Dentistry, Palacky University, Olomouc 77515, Czech Republic

[4]Departments of Experimental Medicine, and Immunology, University Hospital Olomouc 77515, Czech Republic

*** Correspondence:**

Amit Kishore: amitkishore.bio@gmail.com

Martin Petrek: martin.petrek2@fnol.cz

Supplementary Material

Supplementary Table S1: miRNAs involved in alveolar macrophage polarization and their functions in pulmonary fibrosis

**Macrophage derived microRNAs**	**Model**	**Targets**	**Potential immune function**	**Reference**
**I. Macrophage M1**				
↑ miR-21	Mouse	STAT3	Drives M1 and inhibits M2 macrophage polarization	(1)
	BMSCs	TNFα	Inhibit M1 polarization. Low miR-21 levels suggest higher RILI incidence and grade, lower pulmonary function in patients, and enhancement of IL6 and TNFα expressions, especially during the acute phase	(2)
↑ miR-21-5p	Mouse lung I/R model and in vitro H/R model, MSC-Exo	PTEN and PDCD4	Reduced M1 macrophages polarization, lung edema and dysfunction, as well as secretion of HMGB1, IL8, IL1β, IL6, IL17 and TNFα	(3)
↑ miR-33	Mouse	ABCA1 and ABCG1	Elevated proinflammatory cytokines, suppression of anti-inflammatory lipid transporters	(4)
↑ miR-34a	Mouse	*Notch1*	Inhibits production of pro-inflammatory cytokines	(5)
↑ miR-101	Human THP-1-derived macrophages and HepG2 hepatoblastoma cell line	ABCA1	Pro-inflammatory miR-101 negatively regulate the expression of ABCA1 under inflammatory conditions such as IL6 and TNFα	(6)
	Mouse macrophage RAW264.7 cell line	MKP-1	Enhanced LPS-induced pro-inflammatory cytokine production in macrophages via activation of MAPK by targeting MAPK phosphatase-1	(7)
↑ miR-125b-5p	Mouse BMDMs	Not reported	Inhibition of miR-125b-5p mitigates M1 macrophage polarization	(8)
↑ miR-125b	Mouse	IRF4	Enhance pro-inflammatory responses, induced immune responses with increased Ag-specific T cell activation and antitumor immunity	(9)
↑ miR-127-3p	Mouse BMDMs	Not reported	MiR-127-3p promotes M1 signature gene expression	(8)

↑ miR-127	Mouse	Bcl6 and DUSP1	Increased production of proinflammatory cytokines, promote proinflammatory M1 macrophage development through JNK-dependent mechanism	(10)
↑ miR-140	Mouse lung fibroblasts	TGFβ1/Smad3 pathway	Inhibits fibronectin in lung fibroblasts	(11)
↑ miR-146b	Mouse BMDMs	TLR4, MyD88, IRAK1, TRAF6	Anti-inflammatory activity, modulates TLR4 signaling pathway, miR-146b overexpression ameliorates LPS-induced ALI	(12, 13)
↑ miR-148a-3p	Mouse	*Pten*	A novel downstream molecule of Notch signaling to promote the differentiation of monocytes into macrophages in the presence of GM-CSF, enhance M1 macrophage polarization and pro-inflammatory responses through PTEN/AKT-mediated upregulation of NFκB signaling	(14)
↑ miR-155	Mouse	SHIP-1	Significantly reduced fibrosis and EMT in miR-155KO mice involving PI3K/AKT, JAK/STAT3, and SMAD/STAT signaling pathways	(15)
	Mouse and lung fibroblasts	*LXRα*	Reduced exacerbated fibrotic response, profibrotic IPF phenotype and fibroblasts	(16)
	Mouse	SOCS-1	Promote LPS-induced ALI in mice and rats	(17)
	Mouse	IL13Rα1	Development of a healthy immune system and functions as well as in the inflammatory pro-Th1/M1 immune profile	(18)
↑ miR-200b	Mouse	DUSP1	Promotes LPS-induced pro-inflammatory cytokines and injury, pro-inflammatory activities	(19)
↑ miR-200c	Mouse	DUSP1	Promotes LPS-induced pro-inflammatory cytokines and injury, pro-inflammatory activities	(19)
↑ miR-342-5p	Mouse	Bmpr2, Akt1	Induces pro-inflammatory macrophage markers (NOS2 and IL6) in the formation of atherosclerotic lesions	(20)
↑ miR-429	Mouse	DUSP1	Promotes LPS-induced pro-inflammatory cytokines and ALI	(19)
↓ let-7e	Mouse BMDM	MK2	Downregulated let-7e promoted LPS-induced macrophage activation and ALI via MK2 and enhanced CREB activation. The let-7e level is negatively associated with the LPS-induced	(21)

			expression of proinflammatory cytokines TNF-α and IL6, as well as chemokine MIP-2.	
**II. Macrophage M2 (M2a, M2b, M2c and M2d subpopulations)**				
↑ let-7c	Mouse	STAT3	Anti-inflammatory with inhibition of cytokines TNFα, IL6 and IL1β in LPS treated alveolar macrophages by targeting STAT3	(22)
	Mouse	PAK1	EZH2/let-7c/PAK1 axis promotes inflammatory macrophage M1 polarization via NIK-IKK-NF-κB signaling	(23)
	Mouse	C/EBP-δ	Overexpression of let-7c in GM-BMM diminished M1 phenotype expression while promoting polarization to the M2 phenotype	(24)
↑ miR-27a-3p	MSC-EVs	NFKB1	Induced M2 macrophage polarization. MSC-EVs derived miR-27a-3p alleviate ALI and M2 macrophage polarization.	(25)
↑ miR-101*	Mouse and human lung fibroblasts (in vitro)	FZD4, FZD6, and TGFBR1	Mir-101 mitigate pulmonary fibrosis by inhibiting fibroblast proliferation and differentiation via supressing WNT5a/NFATc2 and TGF-/Smad2/3 pathways	(26)
↑ miR-124	Mouse	RELA/p65	Overexpression of miR-124-3p in NC8383 cells and lung tissues significantly suppressed LPS-induced p65 expression and cell apoptosis	(27)
	Mouse	MCP-1	Promotes M2 macrophage polarization, high miR-124 level in lungs prevent LPS-induced myeloperoxidase activity mitigating ALI	(28)
	Mouse and macrophage cell line RAW264.7	STAT3 and TACE	Key mediator for the cholinergic anti-inflammatory action	(29)
	Mouse and macrophage cell line RAW264.7	C/EBPα	M2 phenotype development and maintenance	(30)
↑ miR-125a-5p	Mouse GM-BMM and M-BMM	TNFα	Promotes M2 and suppresses M1 macrophage polarization. Significantly enhance the ability of GM-BMM to ingest apoptotic cells	(31)
↑ miR-135b	Mouse	MAPK6	Downregulation of pro-inflammatory factors and cartilage damage, induces M2 polarization of synovial macrophages	(32)
↑ miR-146a	Mouse	TRAF6, IRAK1 and IRF5	Anti-inflammatory targeting TLR4 signaling, protects from ALI via M2 macrophage activation	(33)

↑ miR-328	Mouse	FAM13A	Progression of pulmonary fibrosis	(34)
↑ miR-511-3p	Human monocytes - primary cells and cell lines	TLR4	Mediates anti-inflammatory cytokines production of TGFβ and glucocorticoids in endotoxin tolerant monocytes	(35)
	CRE-induced mouse model of asthma	Ccl2	Polarizes macrophages toward the M2 phenotype	(36)
↑ miR-378-3p	Mouse and RAW264.7 cells	IL-4R/PI3K/Akt-signaling pathway	Alternative macrophage activation	(37)
↑ miR-223	Mouse	HIF-1α, PDK-1 and PFK	Anti-inflammatory, prevent conversion into M1 macrophage polarization after LPS stimulation	(38)
	Mouse	STAT3	Promotes anti-inflammatory response	(39)
↓ let-7a	Human, mouse, BMDM and lung fibroblasts (in vitro)	c-Myc	Arsenite-induced M2 polarization of macrophages	(40)
↓ miR-29a	In vitro macrophage cell culture model of PMA activated THP1 cells	Not reported	Mir-29a downregulation displayed a profibrotic profile with activation of collagen 1a pathway in BAL alveolar macrophages from IPF patients	(41)
↓ miR-33	Mouse	AMPK-α and PGC-1α	Mir-33 deficiency may be protective against bleomycin induced lung injury and fibrosis	(42, 43)
↓ miR-34a	Mouse	Klf4	Inhibition of miRNA-34a promotes M2 macrophage polarization and improves LPS-induced lung injury	(44)
↓ miR-127	Mouse	Bcl6 and Dusp1	Deletion of miR-127 impaired M1 gene expression and led to a M2-biased response	(10)
↓ miR-140	Mouse	Fibronectin, α-SMA and Smad3	Loss of miR-140 showed increased TGF-β1 signaling myofibroblast in lung fibroblasts. miR-140 is a key protective molecule against RILF through inhibiting myofibroblast differentiation and inflammation	(11)
↓ miR-155	Mouse and IPF lung fibroblasts (in vitro)	*ZNF652*	Increased collagen and TGF-β production,	(16)

	Mouse	IL13Rα1	MiR-155 affects the IL-13-dependent regulation of SOCS1, DC-SIGN, CCL18, CD23, and SERPINE involved in the establishment of M2/pro-Th2 phenotype in macrophages	(18)
↓ miR-185	In vitro macrophage cell culture model of PMA activated THP1 cells	Not reported	MiR-185 downregulation displayed a profibrotic profile with activation of AKT pathway in BAL alveolar macrophages from IPF patients	(41)
miR-320a*	Human and Mouse	TGFBR2 and IGF1R	Important regulator of fibrotic process in ILD	(45)

*Not investigated for macrophage M1/M2 polarization

Abbreviations: ABCA1, ATP-binding cassette transporter A1; Akt1, AKT serine/threonine kinase 1; ALI, acute lung injury; AMPK-α, protein kinase AMP-activated catalytic subunit alpha; Bcl6, BCL6 transcription repressor; BAL, bronchoalveolar lavage fluid; BMDMs/BMM, bone marrow derived macrophages; Bmpr2, bone morphogenetic protein receptor type 2; BMSCs, bone marrow mesenchymal stem cells; C/EBP, CCAAT enhancer binding protein beta; c-Myc, MYC proto-oncogene, bHLH transcription factor; CRE, cockroach extract; DUSP1, dual specificity phosphatase 1; EMT, endothelial-mesenchymal transition; EZH2, enhancer of zeste homolog 2; FAM13A, family with sequence similarity 13 member A; FZD, frizzled class receptor; GM-CSF, granulocyte macrophage colony-stimulating factor; H/R model, hypoxia/reoxygenation model; HIF-1α, hypoxia inducible factor 1 subunit alpha; I/R model, ischemia/reperfusion model; IGF1R, insulin like growth factor 1 receptor l; IL13Rα1, interleukin 13 receptor subunit alpha 1; IPF: Idiopathic pulmonary fibrosis; IRAK1, interleukin 1 receptor associated kinase 1; IRF, interferon regulatory factor 4; KLF4, Kruppel like factor 4; let-7, microRNA lethal-7; LPS, lipopolysaccharide; LXRα, nuclear receptor subfamily 1 group H member 3; MAP, mitogen-activated protein; MAPK, mitogen-activated protein kinase; MAPK6, mitogen-activated protein kinase 6; MCP-1, C-C motif chemokine ligand 2; miR, microRNA; MK2, Mitogen-activated protein kinase (MAPK)-activated protein kinase 2; MKP-1, mitogen-activated protein kinase phosphatase 1; MSC-EVs, Mesenchymal stem cell–derived extracellular vesicles; MyD88, MYD88 innate immune signal transduction adaptor; NFKB1, nuclear factor kappa B subunit 1; Notch1, notch receptor 1; PAK1, p21 (RAC1) activated kinase 1; PAKs, p21-activated kinases; PDCD4, programmed cell death 4; PDK-1, pyruvate dehydrogenase kinase 1; PFK, 6-phosphofructo-2-kinase/fructose-2,6-biphosphatase 3; PGC-1α, PPARG coactivator 1 alpha; PMA, phorbol 12-myristate 13-acetate; PTEN, phosphatase and tensin homolog; RELA/p65, RELA proto-oncogene, NF-kB subunit; RILF, Radiation-induced lung fibrosis; RILI, radiation-induced lung injury; SHIP-1, inositol polyphosphate-5-phosphatase D; Smad3, SMAD family member 3; SOCS, suppressors of cytokine signaling; SOCS-1, suppressor of cytokine signaling 1; STAT3, signal transducer and activator of transcription 3; TACE, TNF-α converting enzyme; TGFBR, transforming growth factor beta receptor; TGFβ1, transforming growth factor beta 1; TLR, Toll-like receptor; TNF, tumour necrosis factor; TRAF6, TNF receptor associated factor 6; ZNF652, zinc finger protein 652; α-SMA, alpha smooth muscle actin.

References:

1. Wang Z, Brandt S, Medeiros A, Wang S, Wu H, Dent A, et al. MicroRNA 21 is a homeostatic regulator of macrophage polarization and prevents prostaglandin E2-mediated M2 generation. *PLoS One* (2015) 10(2):e0115855. doi: 10.1371/journal.pone.0115855. PubMed PMID: 25706647; PubMed Central PMCID: PMCPMC4338261.

2. Bao P, Zhao W, Mou M, Liu X. MicroRNA-21 mediates bone marrow mesenchymal stem cells protection of radiation-induced lung injury during the acute phase by regulating polarization of alveolar macrophages. *Translational Cancer Research* (2020) 9(1):231-9.

3. Li JW, Wei L, Han Z, Chen Z. Mesenchymal stromal cells-derived exosomes alleviate ischemia/reperfusion injury in mouse lung by transporting anti-apoptotic miR-21-5p. *Eur J Pharmacol* (2019) 852:68-76. doi: 10.1016/j.ejphar.2019.01.022. PubMed PMID: 30682335.

4. Barna BP, McPeek M, Malur A, Fessler MB, Wingard CJ, Dobbs L, et al. Elevated MicroRNA-33 in Sarcoidosis and a Carbon Nanotube Model of Chronic Granulomatous Disease. *Am J Respir Cell Mol Biol* (2016) 54(6):865-71. doi: 10.1165/rcmb.2015-0332OC. PubMed PMID: 26641802; PubMed Central PMCID: PMCPMC4942222.

5. Jiang P, Liu R, Zheng Y, Liu X, Chang L, Xiong S, et al. MiR-34a inhibits lipopolysaccharide-induced inflammatory response through targeting Notch1 in murine macrophages. *Exp Cell Res* (2012) 318(10):1175-84. doi: 10.1016/j.yexcr.2012.03.018. PubMed PMID: 22483937.

6. Zhang N, Lei J, Lei H, Ruan X, Liu Q, Chen Y, et al. MicroRNA-101 overexpression by IL-6 and TNF-alpha inhibits cholesterol efflux by suppressing ATP-binding cassette transporter A1 expression. *Exp Cell Res* (2015) 336(1):33-42. doi: 10.1016/j.yexcr.2015.05.023. PubMed PMID: 26033364.

7. Gao Y, Liu F, Fang L, Cai R, Zong C, Qi Y. Genkwanin inhibits proinflammatory mediators mainly through the regulation of miR-101/MKP-1/MAPK pathway in LPS-activated macrophages. *PLoS One* (2014) 9(5):e96741. doi: 10.1371/journal.pone.0096741. PubMed PMID: 24800851; PubMed Central PMCID: PMCPMC4011752.

8. Huang R, Qin C, Wang J, Hu Y, Zheng G, Qiu G, et al. Differential effects of extracellular vesicles from aging and young mesenchymal stem cells in acute lung injury. *Aging (Albany NY)* (2019) 11(18):7996-8014. doi: 10.18632/aging.102314. PubMed PMID: 31575829; PubMed Central PMCID: PMC6781978.

9. Chaudhuri AA, So AY, Sinha N, Gibson WS, Taganov KD, O'Connell RM, et al. MicroRNA-125b potentiates macrophage activation. *J Immunol* (2011) 187(10):5062-8. doi: 10.4049/jimmunol.1102001. PubMed PMID: 22003200; PubMed Central PMCID: PMCPMC3208133.

10. Ying H, Kang Y, Zhang H, Zhao D, Xia J, Lu Z, et al. MiR-127 modulates macrophage polarization and promotes lung inflammation and injury by activating the JNK pathway. *J Immunol* (2015) 194(3):1239-51. doi: 10.4049/jimmunol.1402088. PubMed PMID: 25520401.

11. Duru N, Zhang Y, Gernapudi R, Wolfson B, Lo PK, Yao Y, et al. Loss of miR-140 is a key risk factor for radiation-induced lung fibrosis through reprogramming fibroblasts and macrophages. *Sci Rep* (2016) 6:39572. doi: 10.1038/srep39572. PubMed PMID: 27996039; PubMed Central PMCID: PMC5172237.

12. Curtale G, Mirolo M, Renzi TA, Rossato M, Bazzoni F, Locati M. Negative regulation of Toll-like receptor 4 signaling by IL-10-dependent microRNA-146b. *Proc Natl Acad Sci U S A* (2013) 110(28):11499-504. doi: 10.1073/pnas.1219852110. PubMed PMID: 23798430; PubMed Central PMCID: PMCPMC3710884.

13. He R, Li Y, Zhou L, Su X, Li Y, Pan P, et al. miR-146b overexpression ameliorates lipopolysaccharide-induced acute lung injury in vivo and in vitro. *J Cell Biochem* (2019) 120(3):2929-39. doi: 10.1002/jcb.26846. PubMed PMID: 30500983.

14. Huang F, Zhao JL, Wang L, Gao CC, Liang SQ, An DJ, et al. miR-148a-3p Mediates Notch Signaling to Promote the Differentiation and M1 Activation of Macrophages. *Front Immunol* (2017) 8:1327. doi: 10.3389/fimmu.2017.01327. PubMed PMID: 29085372; PubMed Central PMCID: PMCPMC5650608.

15. Tang H, Mao J, Ye X, Zhang F, Kerr WG, Zheng T, et al. SHIP-1, a target of miR-155, regulates endothelial cell responses in lung fibrosis. *FASEB J* (2020) 34(2):2011-23. doi: 10.1096/fj.201902063R. PubMed PMID: 31907997; PubMed Central PMCID: PMCPMC7449602.

16. Kurowska-Stolarska M, Hasoo MK, Welsh DJ, Stewart L, McIntyre D, Morton BE, et al. The role of microRNA-155/liver X receptor pathway in experimental and idiopathic pulmonary fibrosis. *J Allergy Clin Immunol* (2017) 139(6):1946-56. doi: 10.1016/j.jaci.2016.09.021. PubMed PMID: 27746237; PubMed Central PMCID: PMC5457127.

17. Wang W, Liu Z, Su J, Chen WS, Wang XW, Bai SX, et al. Macrophage micro-RNA-155 promotes lipopolysaccharide-induced acute lung injury in mice and rats. *Am J Physiol Lung Cell Mol Physiol* (2016) 311(2):L494-506. doi: 10.1152/ajplung.00001.2016. PubMed PMID: 27371731.

18. Martinez-Nunez RT, Louafi F, Sanchez-Elsner T. The interleukin 13 (IL-13) pathway in human macrophages is modulated by microRNA-155 via direct targeting of interleukin 13 receptor alpha1 (IL13Ralpha1). *J Biol Chem* (2011) 286(3):1786-94. doi: 10.1074/jbc.M110.169367. PubMed PMID: 21097505; PubMed Central PMCID: PMCPMC3023473.

19. Xiao J, Tang J, Chen Q, Tang D, Liu M, Luo M, et al. miR-429 regulates alveolar macrophage inflammatory cytokine production and is involved in LPS-induced acute lung injury. *Biochem J* (2015) 471(2):281-91. doi: 10.1042/BJ20131510. PubMed PMID: 26431850.

20. Wei Y, Nazari-Jahantigh M, Chan L, Zhu M, Heyll K, Corbalan-Campos J, et al. The microRNA-342-5p fosters inflammatory macrophage activation through an Akt1- and microRNA-155-dependent pathway during atherosclerosis. *Circulation* (2013) 127(15):1609-19. doi: 10.1161/CIRCULATIONAHA.112.000736. PubMed PMID: 23513069.

21. Wu Y, He H, Ding Y, Liu S, Zhang D, Wang J, et al. MK2 mediates macrophage activation and acute lung injury by regulating let-7e miRNA. *Am J Physiol Lung Cell Mol Physiol* (2018) 315(3):L371-L81. doi: 10.1152/ajplung.00019.2018. PubMed PMID: 29770701.

22. Yu J-H, Long L, Luo Z-X, Li L-M, You J-R. Anti-inflammatory role of microRNA let-7c in LPS treated alveolar macrophages by targeting STAT3. *Asian Pacific Journal of Tropical Medicine* (2016) 9(1):72-5. doi: https://doi.org/10.1016/j.apjtm.2015.12.015.

23. Zhang W, Liu H, Liu W, Liu Y, Xu J. Polycomb-mediated loss of microRNA let-7c determines inflammatory macrophage polarization via PAK1-dependent NF-kappaB pathway. *Cell Death Differ* (2015) 22(2):287-97. doi: 10.1038/cdd.2014.142. PubMed PMID: 25215948; PubMed Central PMCID: PMCPMC4291490.

24. Banerjee S, Xie N, Cui H, Tan Z, Yang S, Icyuz M, et al. MicroRNA let-7c regulates macrophage polarization. *J Immunol* (2013) 190(12):6542-9. doi: 10.4049/jimmunol.1202496. PubMed PMID: 23667114; PubMed Central PMCID: PMC3679284.

25. Wang J, Huang R, Xu Q, Zheng G, Qiu G, Ge M, et al. Mesenchymal Stem Cell-Derived Extracellular Vesicles Alleviate Acute Lung Injury Via Transfer of miR-27a-3p. *Crit Care Med* (2020) 48(7):e599-e610. doi: 10.1097/CCM.0000000000004315. PubMed PMID: 32317602.

26. Huang C, Xiao X, Yang Y, Mishra A, Liang Y, Zeng X, et al. MicroRNA-101 attenuates pulmonary fibrosis by inhibiting fibroblast proliferation and activation. *J Biol Chem* (2017) 292(40):16420-39. doi: 10.1074/jbc.M117.805747. PubMed PMID: 28726637; PubMed Central PMCID: PMCPMC5633105.

27. Liang Y, Xie J, Che D, Zhang C, Lin Y, Feng L, et al. MiR-124-3p helps to protect against acute respiratory distress syndrome by targeting p65. *Biosci Rep* (2020) 40(5). doi: 10.1042/BSR20192132. PubMed PMID: 32391561; PubMed Central PMCID: PMCPMC7253404.

28. Gu W, Yao L, Li L, Zhang J, Place AT, Minshall RD, et al. ICAM-1 regulates macrophage polarization by suppressing MCP-1 expression via miR-124 upregulation. *Oncotarget* (2017) 8(67):111882-901. doi: 10.18632/oncotarget.22948. PubMed PMID: 29340098; PubMed Central PMCID: PMCPMC5762366.

29. Sun Y, Li Q, Gui H, Xu DP, Yang YL, Su DF, et al. MicroRNA-124 mediates the cholinergic anti-inflammatory action through inhibiting the production of pro-inflammatory cytokines. *Cell Res* (2013) 23(11):1270-83. doi: 10.1038/cr.2013.116. PubMed PMID: 23979021; PubMed Central PMCID: PMCPMC3817544.

30. Veremeyko T, Siddiqui S, Sotnikov I, Yung A, Ponomarev ED. IL-4/IL-13-dependent and independent expression of miR-124 and its contribution to M2 phenotype of monocytic cells in normal conditions and during allergic inflammation. *PLoS One* (2013) 8(12):e81774. doi: 10.1371/journal.pone.0081774. PubMed PMID: 24358127; PubMed Central PMCID: PMCPMC3864800.

31. Banerjee S, Cui H, Xie N, Tan Z, Yang S, Icyuz M, et al. miR-125a-5p regulates differential activation of macrophages and inflammation. *J Biol Chem* (2013) 288(49):35428-36. doi: 10.1074/jbc.M112.426866. PubMed PMID: 24151079; PubMed Central PMCID: PMCPMC3853290.

32. Wang R, Xu B. TGF-beta1-modified MSC-derived exosomal miR-135b attenuates cartilage injury via promoting M2 synovial macrophage polarization by targeting MAPK6. *Cell Tissue Res* (2021). doi: 10.1007/s00441-020-03319-1. PubMed PMID: 33404840.

33. Vergadi E, Vaporidi K, Theodorakis EE, Doxaki C, Lagoudaki E, Ieronymaki E, et al. Akt2 deficiency protects from acute lung injury via alternative macrophage activation and miR-146a induction in mice. *J Immunol* (2014) 192(1):394-406. doi: 10.4049/jimmunol.1300959. PubMed PMID: 24277697.

34. Yao MY, Zhang WH, Ma WT, Liu QH, Xing LH, Zhao GF. microRNA-328 in exosomes derived from M2 macrophages exerts a promotive effect on the progression of pulmonary fibrosis via FAM13A in a rat model. *Exp Mol Med* (2019) 51(6):1-16. doi: 10.1038/s12276-019-0255-x. PubMed PMID: 31164635; PubMed Central PMCID: PMC6547742.

35. Curtale G, Renzi TA, Drufuca L, Rubino M, Locati M. Glucocorticoids downregulate TLR4 signaling activity via its direct targeting by miR-511-5p. *Eur J Immunol* (2017) 47(12):2080-9. doi: 10.1002/eji.201747044. PubMed PMID: 28776644.

36. Do DC, Mu J, Ke X, Sachdeva K, Qin Z, Wan M, et al. miR-511-3p protects against cockroach allergen-induced lung inflammation by antagonizing CCL2. *JCI Insight* (2019) 4(20). doi: 10.1172/jci.insight.126832. PubMed PMID: 31536479; PubMed Central PMCID: PMCPMC6824444.

37. Ruckerl D, Jenkins SJ, Laqtom NN, Gallagher IJ, Sutherland TE, Duncan S, et al. Induction of IL-4Ralpha-dependent microRNAs identifies PI3K/Akt signaling as essential for IL-4-driven murine macrophage proliferation in vivo. *Blood* (2012) 120(11):2307-16. doi: 10.1182/blood-2012-02-408252. PubMed PMID: 22855601; PubMed Central PMCID: PMCPMC3501641.

38. Dang CP, Leelahavanichkul A. Over-expression of miR-223 induces M2 macrophage through glycolysis alteration and attenuates LPS-induced sepsis mouse model, the cell-based therapy in sepsis. *PLoS One* (2020) 15(7):e0236038. doi: 10.1371/journal.pone.0236038. PubMed PMID: 32658933; PubMed Central PMCID: PMCPMC7357756.

39. Chen Q, Wang H, Liu Y, Song Y, Lai L, Han Q, et al. Inducible microRNA-223 down-regulation promotes TLR-triggered IL-6 and IL-1beta production in macrophages by targeting STAT3. *PLoS One* (2012) 7(8):e42971. doi: 10.1371/journal.pone.0042971. PubMed PMID: 22937006; PubMed Central PMCID: PMCPMC3427313.

40. Xiao T, Zou Z, Xue J, Syed BM, Sun J, Dai X, et al. LncRNA H19-mediated M2 polarization of macrophages promotes myofibroblast differentiation in pulmonary fibrosis induced by arsenic exposure. *Environ Pollut* (2021) 268(Pt A):115810. doi: 10.1016/j.envpol.2020.115810. PubMed PMID: 33162208.

41. Tsitoura E, Wells AU, Karagiannis K, Lasithiotaki I, Vasarmidi E, Bibaki E, et al. MiR-185/AKT and miR-29a/collagen 1a pathways are activated in IPF BAL cells. *Oncotarget* (2016) 7(46):74569-81. doi: 10.18632/oncotarget.12740. PubMed PMID: 27769060; PubMed Central PMCID: PMCPMC5342687.

42. Zhao F, Madenspacher J, Lin W-C, Fessler MB. MicroRNA-33 Regulates Inflammation and Fibrosis in the Injured Lung. *A60 LUNG INJURY, SEPSIS, AND ARDS*. (2019). p. A2107-A.

43. Ahangari F, Chioccioli M, Malik S, Baernthaler T, Price NL, Ding S, et al. Macrophage Specific Regulatory Role of miR-33 in Pulmonary Fibrosis. *TP116 TP116 PRECLINICAL THERAPEUTIC MODELS OF FIBROSIS*. American Thoracic Society International Conference Abstracts. American Thoracic Society (2021). p. A4452-A.

44. Khan MJ, Singh P, Dohare R, Jha R, Rahmani AH, Almatroodi SA, et al. Inhibition of miRNA-34a Promotes M2 Macrophage Polarization and Improves LPS-Induced Lung Injury by Targeting Klf4. *Genes (Basel)* (2020) 11(9). doi: 10.3390/genes11090966. PubMed PMID: 32825525; PubMed Central PMCID: PMC7563942.

45. Li Y, Huang J, Hu C, Zhou J, Xu D, Hou Y, et al. MicroRNA-320a: an important regulator in the fibrotic process in interstitial lung disease of systemic sclerosis. *Arthritis Res Ther* (2021) 23(1):21. doi: 10.1186/s13075-020-02411-9. PubMed PMID: 33430962; PubMed Central PMCID: PMCPMC7802184.
